# Autoantigen TRIM21/Ro52 as a Possible Target for Treatment of Systemic Lupus Erythematosus

**DOI:** 10.1155/2012/718237

**Published:** 2012-06-04

**Authors:** Ryusuke Yoshimi, Yoshiaki Ishigatsubo, Keiko Ozato

**Affiliations:** ^1^Department of Internal Medicine and Clinical Immunology, Yokohama City University Graduate School of Medicine, Yokohama 236-0004, Japan; ^2^Program in Genomics of Differentiation, National Institute of Child Health and Human Development, National Institutes of Health, Bethesda, MD 20892, USA

## Abstract

Systemic lupus erythematosus (SLE) is a chronic, systemic, and autoimmune disease, whose etiology is still unknown. Although there has been progress in the treatment of SLE through the use of glucocorticoid and immunosuppressive drugs, these drugs have limited efficacy and pose significant risks of toxicity. Moreover, prognosis of patients with SLE has remained difficult to assess. TRIM21/Ro52/SS-A1, a 52-kDa protein, is an autoantigen recognized by antibodies in sera of patients with SLE and Sjögren's syndrome (SS), another systemic autoimmune disease, and anti-TRIM21 antibodies have been used as a diagnostic marker for decades. TRIM21 belongs to the tripartite motif-containing (TRIM) super family, which has been found to play important roles in innate and acquired immunity. Recently, TRIM21 has been shown to be involved in both physiological immune responses and pathological autoimmune processes. For example, TRIM21 ubiquitylates proteins of the interferon-regulatory factor (IRF) family and regulates type I interferon and proinflammatory cytokines. In this paper, we summarize molecular features of TRIM21 revealed so far and discuss its potential as an attractive therapeutic target for SLE.

## 1. Introduction

Systemic lupus erythematosus (SLE) is a chronic, systemic, and autoimmune disease of unknown etiology. The disease is characterized by the presence of a variety of autoantibodies in patients' sera, including those specific for nuclear antigens called antinuclear antibodies (ANA). Although current therapies of SLE largely rely on glucocorticoids and immunosuppressant drugs, the efficacy is limited so far and there hold significant risks of toxicity. In order to better understand the pathogenesis and find new therapeutic designs, a systemic characterization of the molecular and cellular basis of signaling abnormalities in the immune system that lead to autoimmune response and inflammation is urgently required.

In sera of patients with SLE and Sjögren's syndrome (SS), another systemic autoimmune disease, an ANA specific for an autoantigen called Ro (also called SS-A) has been known since the 1960s [1–3]. While anti-Ro antibodies are found primarily in patients with SLE and SS, they are also sometimes seen in other systemic autoimmune diseases, such as systemic sclerosis, polymyositis/dermatomyositis, mixed connective tissue disease, and rheumatoid arthritis [4, 5]. Although these anti-Ro antibodies have been used as a useful diagnostic marker for SLE and SS for decades, the molecular identity of Ro autoantigen had not been clarified for a long time. Later, Ro antigen was found to consist of two proteins, Ro52 (also called TRIM21, SSA1, or RNF81) and Ro60 (also called SSA2 or TROVE2), which seem to have functions not directly related to each other [6–8].

Anti-Ro antibodies have been reported to be associated with photosensitivity, subacute cutaneous lupus, cutaneous vasculitis, hematological disorder, interstitial lung disease, and neonatal lupus including congenital heart block [5, 9–15]. The anti-Ro antibodies are associated with the HLA-DR3 and/or DR2 haplotype and C4 complement component deficiency [16, 17]. Among the anti-Ro antibodies, anti-TRIM21/Ro52 antibodies have been used for diagnosis and monitoring and have been shown to act as a pathological factor [18, 19].

The *TRIM21* gene has also been linked to the diseases, although the sample numbers were small in the reported studies. These studies suggest possible associations between allelic polymorphisms of *TRIM21* and the disease susceptibility and increased anti-TRIM21 antibodies in SLE and SS (Table 1). The rs660 C/T single-nucleotide polymorphism (SNP) has been shown to be associated with SLE in African Americans [20, 21]. In this SNP, population size of rs660C/C in patients with SLE was around one ninth of that in healthy controls. The rs5030767 C/T, rs5030768 A/G, rs915956 C/T, and rs4144331 C/A SNPs were shown to be associated with anti-TRIM21 antibody-positive primary SS in a Norwegian population, among which rs915956 shows the strongest association [22]. Population size of rs915956C/C in anti-TRIM21 antibody-positive patients with SS was about one half of that in healthy controls. The rs7947461 A/G SNP is one of genetic factors involved in Japanese SS [23]. rs7947461A/A population in anti-TRIM21 antibody-positive patients with SS was six times as large as that in anti-TRIM21 antibody-negative patients.

Furthermore, about a twofold increase in the expression of TRIM21 transcripts in peripheral blood mononuclear cells (PBMC) of patients with SLE and SS as compared with healthy controls has been reported, although data on protein levels was not shown [24]. Thus, not only the antibodies to TRIM21 but also the TRIM21 antigen itself has been suggested to have a role, in some way, in the pathogenesis of SLE and SS [25]. In this paper, we focus on the nature and function of the TRIM21 molecule and discuss its potential as a therapeutic target of systemic autoimmune diseases.

## 2. The TRIM21 Gene Locus, Molecular Structure, and Expression Pattern

In 1991, two independent groups cloned the cDNA of human TRIM21, a 475-aa and 52-kDa protein [26, 27]. Subsequently, the human *TRIM21 *gene was mapped to the region 11p15.5 on chromosome 11 and the exon-intron structure was clarified [20, 28, 29]. The murine homologue, *Trim21*, has also been cloned [30]. The amino acid sequence identity between the murine and the human TRIM21 proteins is 69.9%, and when conservative substitutions were taken into account, the similarly increases to 81.5%. The identity is much higher in regions containing predicted functional domains [30]. The *TRIM21* gene is 8.8 kb (in human) or 7.5 kb (in mouse) in size, consisting of 7 (in human) or 8 (in mouse) exons with conserved exon-intron structure. There are two large regions of closely related *TRIM *gene clusters in the human and murine genomes [31]. First, *TRIM10*, *TRIM15*, *TRIM26*, *TRIM31*,* TRIM39*, and* TRIM40* are located in the major histocompatibility complex region on chromosome 6 in human and 17 in mouse. Second, *TRIM3*, *TRIM5*, *TRIM6*, *TRIM21*, *TRIM22*, *TRIM34*, *TRIM66*, and* TRIM68* are located on chromosome 11 in human and 7 in mouse.

The TRIM21 protein belongs to the tripartite motif-containing (TRIM) superfamily by its molecular structure [32, 33]. A common structural feature of the TRIM superfamily is the highly conserved RBCC motif, containing the RING, B-box, and coiled-coil domains in this order [31, 34]. TRIM proteins exhibit a wide range of activities, including the regulation of innate and adaptive immunity [33]. Interestingly, TRIM68, encoded by a gene that is adjacent to *TRIM21*, has a structure that is similar to TRIM21. TRIM68 is another autoantigen, SS-56, found in patients with SLE and SS [35]. The N terminus of TRIM21 has the RING domain (aa 15–58 in human) with two Zn^2+^-binding sites and the B-box domain (aa 91–128) with one Zn^2+^-binding site [26]. The middle part of the protein, which includes the leucine zipper, has a coiled-coil structure (aa 125–235). The C terminus of TRIM21 contains a PRYSPRY domain (aa 286–465; also referred to as B30.2) also found in many other TRIM proteins [36, 37].

TRIM21 is expressed ubiquitously as determined by *in situ *hybridization [32, 38–41]. Recently, more precise distribution patterns were analyzed for TRIM21 using *Trim21*-null mice with an enhanced green fluorescent protein (EGFP) reporter gene by two independent groups [42, 43]. TRIM21 is expressed at the highest levels in immune organs, such as lymph nodes, spleen and thymus in the mice. Among leukocyte subsets, TRIM21 expression is generally high in T cells, natural killer T cells, and macrophages and dendritic cells, but lower in B cells and granulocytes, although the levels varied for cells in each type [42]. For example, among B cell subsets, B-1a cells expressed TRIM21 levels twice that of plasma cells. Within dendritic cell (DC) subsets, TRIM21 levels were higher in the conventional DCs (cDCs) than plasmacytoid DCs (pDCs).

TRIM21 expression levels vary substantially during the early development of both the B and T cell lineages [42]. As for intracellular localization, TRIM21 mostly resides in the cytoplasm, although low levels are also found in the nucleus [44, 45]. A study with overexpressed recombinant GFP-TRIM21 fusion protein showed that full-length TRIM21 is almost exclusively localized in the cytoplasm while deletion mutants lacking the coiled-coil domain spread evenly throughout the cell, suggesting that the cytoplasmic localization of TRIM21 is mediated by the coiled-coil region [46]. In the cytoplasm, TRIM21 has been shown to localize in unidentified distinct structures called “cytoplasmic body,” which is located along the microtubule network [32, 47, 48].

TRIM21 has been extensively documented as an interferon- (IFN-) inducible protein in various cell types [41–43, 47, 49–52]. TRIM21 is also induced by viral infection or Toll-like receptor (TLR) engagement via type I IFN induction [42, 50, 53]. TRIM21 is translocated from the cytoplasm into the nucleus upon IFN-*α* stimulation [47, 50]. On the other hand, TRIM21 translocation in the cell is also mediated by other stimulations. It has been reported that TRIM21 translocates from the cytoplasm to cell surface in apoptotic or stressed cells. TRIM21 is translocated to apoptotic blebs and associated with the plasma membrane in apoptotic endothelial cells [54]. A similar translocation has also been demonstrated in apoptotic fetal cardiomyocytes [55, 56]. In keratinocytes, TRIM21 is translocated from the cytoplasm to the cell surface in response to UV light, oxidative stress, nitric oxide (NO) or estradiol treatment [57–62]. TRIM21 expression has also been reported to be elevated in the brain microvasculature, which may be related to brain vascular involvement in autoimmune diseases [63].

TRIM21 has an alternative splicing variant, named Ro52*β*. The entire exon 4, comprising the C-terminal part of the coiled-coil domain, is spliced off in Ro52*β*. So far Ro52*β* has been detected only at the mRNA level and to date there is no report showing the Ro52*β* protein, with the predicted molecular weight of 45 kDa [28, 64]. Ro52*β* transcript levels are markedly increased in human fetal hearts between 14 weeks and 16 weeks of gestation and diminish at 22–25 weeks [64]. The expression levels of the two splicing variants have been investigated also in salivary glands of patients with SS, but no significant difference in the levels of either variant between patients and healthy controls has been detected in this paper [65].

## 3. TRIM21 Functions

The RING domains of TRIM family members have been shown to have E3 ubiquitin ligase activity, which mediates ubiquitylation events [33]. Following the first demonstration of TRIM21 ubiquitin E3 ligase activity by Wada et al., several reports supporting the conclusion have been published by other groups [24, 38, 41, 66].

There have been many papers reporting TRIM21-interacting proteins (Table 2). IFN regulatory factors (IRFs), a family of transcription factors that stimulates transcription of type I IFN and other immune response genes after activation, have been shown to be substrates of the TRIM21 ubiquitin ligase. TRIM21 interacts with IRF8, following stimulation by IFN and TLR ligands, suggesting a role for this protein in innate immunity [41]. This interaction, which occurs in the nucleus, mediates IRF8 ubiquitylation and increases the ability of IRF8 to promote interleukin (IL)-12p40 transcription in murine macrophage cell line. Later, sequestosome 1 (also called p62), an adaptor protein with multiple functions, was also shown to interact with TRIM21 and IRF8, which leads to increased polyubiquitylation and destabilization of IRF8 in stimulated macrophages, resulting in attenuation of IL-12p40 expression [67]. There are two contradictory reports as to the role of TRIM21 in IRF3 ubiquitylation. One group reported that TRIM21 ubiquitylates IRF3 following TLR3 or TLR4 stimulation, which leads to its degradation at proteasome, resulting in negative regulation of type I IFN [68]. Another group showed that TRIM21 stabilizes IRF3 by interfering with the interaction between peptidyl-prolyl cis/trans isomerase, NIMA-interacting 1 (Pin1), and IRF3, thus enhancing the strength and duration of primary antiviral response [69]. TRIM21-mediated IRF7 ubiquitylation promotes the degradation of IRF7 following TLR7 or TLR9 stimulation, resulting in a decrease of IFN-*α* production [70]. It is also reported that Fas-associated death domain (FADD) and TRIM21 cooperatively ubiquitylate IRF7, interfere with the ubiquitin ligase activity of TNF receptor-associated factor 6 (TRAF6) by affecting phosphorylation status of IRF7, and repress IFN-*α* activation in Sendai virus-infected cells [71]. Characterization of immune cells from TRIM21-deficient mice demonstrated that TRIM21 is required for polyubiquitylation and degradation of IRF5 in addition to IRF3, IRF7, and IRF8, although interaction between TRIM21 and IRF5 has not shown yet [42, 43, 70]. In sum, although the role of TRIM21 in regulating the IFN pathway is still controversial, multiple lines of evidence strongly suggest that TRIM21 is an important regulator in innate immune responses.

TRIM21 may also have important roles in acquired immunity. TRIM21 may regulate T-cell activation or proliferation, since overexpression of TRIM21 has been shown to increase IL-2 production in CD28-stimulated Jurkat T cells [72]. Reduced IL-2 results in the suppression of activation-induced cell death and increased longevity of autoreactive T cells in patients with SLE [73]. In a murine B-cell line, TRIM21 negatively regulates cell growth in steady state and increases apoptotic cell death after activation via the CD40 pathway [24]. Based on the antigen-driven immune response hypothesis, the produced apoptotic cell debris, which could be the source of TRIM21 autoantigen, may induce the production of anti-TRIM21 antibodies [74]. These autoantibodies could cause the subsequent inflammatory condition and create a positive feedback amplification loop of inflammation. Additionally, it also suggests that the increased expression of the TRIM21 autoantigen in patients may be directly involved in the reduced cellular proliferation and increased apoptotic cell death observed in SLE and SS.

Another protein that has been well investigated as a TRIM21-binding protein is IgG [75–80]. The interaction was first suspected from the unexpected observation that normal human sera could precipitate TRIM21 [81]. Then several groups reported that TRIM21 binds to the Fc region of human IgG_1_, IgG_2_, and IgG_4_ via the PRYSPRY domain with high affinity (*Kd* = 37 nM) [76, 78, 80]. The PRYSPRY domain binds to different parts of the IgG Fc region from where Fc*γ*R binds to. On the other hand, human TRIM21 does not seem to interact with murine IgG [80]. Although the physiological meaning of TRIM21-IgG interactions remains unknown, it may play a role in the pathogenesis of SLE and SS. Recently, Mallery et al. reported that TRIM21 rapidly recruits incoming antibody-coated virus and targets it to the proteasome via its E3 ubiquitin ligase activity, resulting in degradation of virions in the cytosol [82]. Infection experiments demonstrate that at physiological antibody concentrations TRIM21 neutralizes viral infection. Based on these data, the authors suggest a new interesting concept that antibodies mediate an intracellular immune response and protect cell interior and that TRIM21 acts as an intracellular antibody receptor for the ubiquitin-proteasome system [83]. Nevertheless, it remains unclear how the proteasome deals with such a large substrate as the viral capsid.

TRIM21 has been shown to interact with several additional proteins. The TRIM21-Skp2-SKp1-Cul1 complex ubiquitylates and degrades the cyclin-dependent kinase inhibitor p27 and promotes S-phase progression in human cell line [38]. TRIM21 associates with the apoptosis-related proteins, Daxx and FLASH, and induces cytoplasmic localization of Daxx in cooperation with FLASH [84]. TRIM21 interacts with tribbles homolog 2 (TRIB2) and leads to degradation of a differentiation-inducing transcription factor CCAAT/enhancer-binding protein *α* (C/EBP*α*), causing lung tumorigenesis [85]. TRIM21 binds to and activates decapping enzyme 2 (DCP2), suggesting a role in mRNA metabolism in response to cellular stimulation [86].

The rs660 polymorphism in *TRIM21* correlates with the age at which patients with sickle cell disease become alloimmunized following red blood cell (RBC) transfusion [87]. Because this polymorphism is found outside of the *TRIM21-*coding regions, it has been proposed to play a role in regulating levels of TRIM21 expression [20, 87]. Thus, it has been hypothesized that rs660C/T leads to lower levels of TRIM21 expression than rs660T/T, resulting in reduced negative feedback responses and higher rates of alloimmunization against transfused RBCs. However, depletion of TRIM21 expression did not enhance transfusion-induced humoral alloimmunization in murine model [88].

How do autoantibodies to TRIM21 in sera affect the function of TRIM21 inside cells? First, it should be clarified how autoantibodies meet intracellular TRIM21 molecules. Recently, it has been reported that IgG can enter the cytoplasm of nonimmune cells through the cell membrane together with virus [82]. This suggests the possibility of intracellular autoantigen-autoantibody interaction. Second, we should know the effect of autoantigen-autoantibody interaction on the molecular function of TRIM21. A recent report shows that anti-TRIM21 autoantibodies inhibit the E3 ligase activity of TRIM21 by sterically blocking the E2/E3 interaction between TRIM21 and UBE2E1 [89]. Although it still remains to be investigated whether enough anti-TRIM21 autoantibodies can enter cells to inhibit TRIM21 function sufficiently, this inhibition may contribute to the pathogenesis of SLE and SS by inhibiting TRIM21-mediated ubiquitylation.

## 4. *Trim21*-Knockout Mice

Two groups have recently disrupted the murine *Trim21 *gene for analyses of TRIM21 function [42, 43]. They reported markedly different phenotypes for their mutant mice, although there are some similarities. The *Trim*21^−/−^ mice described by Yoshimi et al. live a normal life span with no overt abnormal phenotypes [42]. Their immune cells show normal composition and elicit comparable responses to pathogen signals and antigen stimulation as cells of wild-type mice. In addition, Yoshimi et al. noted about a twofold increase in production of NF-*κ*B-dependent cytokines, such as IL-1*β*, TNF*α*, and IL-6, was observed in *Trim*21^−/−^ fibroblasts as compared to wild-type fibroblasts. Data consistent with this paper were published by another group [90, 91]. That is, TRIM21 downregulates NF-*κ*B signalling by monoubiquitylating I*κ*B kinase *β* (IKK*β*) and translocating IKK*β* to autophagosomes. Several *Trim *genes,* Trim12*, *Trim30*, or *Trim34*, encoded near *Trim21 *were found upregulated in *Trim*21^−/−^ cells, suggesting that the absence of TRIM21 protein in these mice was compensated by a network of cross-talk among related TRIM members.

On the other hand, Espinosa et al. reported that TRIM21-deficient mice develop uncontrolled inflammation and systemic autoimmunity as a consequence of minor tissue injury caused by ear tagging [43]. However the mutant mice still carried a C-terminally truncated *Trim21* and expressed the corresponding transcript [92, 93]. In these mice, the general autoimmune pathology and neutrophil recruitment to the site of injury were IL-23 dependent, as they were not observed when the mice were crossed to IL-23p19-deficient mice. Bone-marrow-derived macrophages and splenocytes from the mutant mice released more inflammatory cytokines, IL-6, TNF*α*, type I IFN, and IL-23, upon TLR activation as compared to wild type. Overall, these data demonstrate that TRIM21 is induced by IFN activation of immune cells, where it acts as a negative regulator of IFN signaling.

Although the possibility that differences in environmental factors between the colonies of the two laboratories could account for the discrepancies cannot be ruled out, there are marked differences in the gene disruption method used by the two groups [92, 93]. First, in mice made by Espinosa et al. the overexpressed truncated protein could act as a dominant negative mutant, interfering with the function of a number of TRIM family members, including the normal TRIM21, and resulting in a stronger phenotype than the null mutant reported by Yoshimi et al. Second, the mutant mice reported by Espinosa et al. did not show a compensatory increase in the expression of other *Trim* genes as observed in the null mutant, suggesting that the stronger phenotype is due to a lack of the compensatory mechanisms. Anyway, the results from these two mutant mice suggest that TRIM21 is a negative regulator for proinflammatory cytokine production. Further experiments using animal disease models are needed for pursuing the proposed use of TRIM21 as a therapeutic target in the future. For example, it will be interesting to investigate whether disease severity of murine lupus model strains, such as MRL/*lpr*, BWF1, and B6-*Yaa*, can be diminished by adenovirus-mediated *Trim21 *gene transfer.

## 5. TRIM21 as a Possible Therapeutic Target for SLE

Patients with SLE are usually treated with nonsteroidal anti-inflammatory drugs (NSAIDs), glucocorticoids, antimalarials, and immunosuppressants, including cyclophosphamide, azathioprine, mycophenolate mofetil, cyclosporine, tacrolimus, and methotrexate. Owing to the use of these drugs, the prognosis of patients with SLE has dramatically improved in the past. Nonetheless, rheumatologists sometimes encounter cases in which it is difficult to control the disease activity or to continue the treatment due to serious side effects.

In patients with SLE, increased expression of a subset of IFN-inducible genes, collectively called “interferon signature,” is often observed [94]. Thus, the high level of type I IFN and IFN-stimulated genes has been suspected to be involved in the pathogenesis of SLE. pDCs produce a large amount of type I IFN via the activation of TLR7 and TLR9 upon viral infection, and these cells are thought to be the main source of IFN-*α* in patients with SLE [95, 96]. While the number of pDC is reduced in the peripheral blood, many pDCs infiltrate into the skin and renal lesions in patients with SLE [97]. The serum levels of IFN are high in a subset of SLE patients and correlate with the disease activity [98]. IFN therapy of hepatitis C can lead to SLE as a side effect [99]. Thus suppression of IFN signaling can be a hopeful strategy for SLE treatment. TRIM21 negatively controls type I IFN production via ubiquitylation of IRFs as discussed above. Because TRIM21 expression is increased by type I IFN, the role of TRIM21 as a negative regulator increases late after inflammation. Thus, TRIM21 could repress and turn off type I IFN production, contributing to protection from prolonged overproduction of type I IFN and the pathogenesis of SLE. Therefore, increasing E3 activity of TRIM21 in type IFN-producing cells, such as pDCs and fibroblasts, may also be a useful strategy for the treatment of SLE.

NF-*κ*B plays a pivotal role in inflammation through its ability to induce transcription of proinflammatory cytokines, chemokines, and adhesion molecules [100]. In SLE, for example, *in situ *expression of activated NF-*κ*B is increased in glomerular endothelial cells, mesangial cells, and infiltrating cells in class IV lupus nephritis, along with upregulation of TNF*α*, IL-1*β*, IL-6, and ICAM-1 expression [101]. Some of the effects of glucocorticoids used in SLE treatment are mediated through the inhibition of NF-*κ*B activation. The NF-*κ*B activation is mediated by IKK via phosphorylation of I*κ*B. TRIM21 downregulates NF-*κ*B signalling by monoubiquitylating IKK*β*, a subunit of IKK complex, and translocating IKK*β* to autophagosomes [42, 90, 91]. Because TRIM21 expression is increased by type I IFN as noted above, the activity of TRIM21 in inhibiting NF-*κ*B signalling can augment at a late phase in inflammation. Thus, TRIM21 could have an essential physiological role in turning off the NF-*κ*B signalling, avoiding prolonged overproduction of proinflammatory cytokines, chemokines, and adhesion molecules. Therefore, a strategy for increasing E3 activity of TRIM21 in macrophages and in many cell types may also be useful for the treatment of SLE.

TRIM21 is substantially induced by type I IFN, type II IFN, and TLR ligands in DCs, macrophages, and fibroblasts. This implies the physiological significance of TRIM21 in an inflammatory state in these cells. Thus, increasing the expression of TRIM21 in these cells can be of advantage to the patients. Additionally, T cells may be an important subset for the therapy targeting TRIM21, because TRIM21 is highly expressed in T cells among leukocyte subsets, suggesting the significance of TRIM21 in T cells. Serum IL-2 levels are reduced in patients with SLE, resulting in the suppression of activation-induced cell death and increased life-span of autoreactive T cells [73]. This also likely causes an increased risk of infection, one of the major prognosis factors for SLE [73]. Because TRIM21 seems to positively regulate IL-2 production by T cells [72], increasing TRIM21 activity in T cells may be a strategy worthy of consideration.

Differentiation of T-cell subsets is biased in patients with SLE. Increased IL-6 production by antigen presenting cells and decreased IL-2 secretion by T cells inhibit the development of regulatory T cells and promote the differentiation of IL-17-producing T (Th17) cells. Given that Th17 cells are found not only in the peripheral blood but also in the inflamed kidney in patients with SLE [102, 103], blockade of IL-17 or IL-23, which is important for IL-17 production by Th17 cells, may help to control SLE pathogenesis. As TRIM21 can negatively regulate IL-23-Th17 pathway [43], increased E3 activity by TRIM21 may contribute to the regulation of these cytokines.

In order to suppress aberrant antibody production, modulating the number and the function of B cells has been regarded as a promising means for controlling SLE. Although small studies and case series using a chimeric anti-CD20 antibody, rituximab, in patients with SLE have initially shown promising data [104–106], a placebo-controlled randomized trial of rituximab in patients with moderate-to-severe SLE failed to observe efficacy of rituximab [107]. Belimumab, a human monoclonal antibody that neutralizes a B-cell survival factor, B-lymphocyte stimulator (BLyS), has received an FDA approval for the treatment of patients with active, autoantibody-positive SLE receiving standard therapy [108]. Because TRIM21 is suggested to negatively regulate B-cell growth and increase apoptotic cell death [24], it may be effective as a B-cell-based treatment of SLE. However, there is also a possibility that increased apoptotic death in B cells may exacerbate the pathogenesis of SLE. On the other hand, increased IL-6 promotes antibody production in humans and mice with lupus [109]. A monoclonal antibody against the IL-6 receptor, tocilizumab, has been reported to be promising in a phase I clinical trial [110]. TRIM21 may suppress antibody production by suppressing IL-6 production.

In sum, increasing the activity of TRIM21 could be beneficial in controlling many aspects of SLE disease activity. Although altering the broad range of activity by TRIM21 could suggest potential risk of a variety of side effects, TRIM21-targeting therapy may provide a new avenue of SLE treatment, which may offer multiple benefits unattainable by existing therapies.

## 6. Conclusions

SLE is an autoimmune disease, in which immune system aberrations as well as heritable, hormonal, and environmental factors contribute to the manifestation of organ damage. Recent data suggest that the autoantigen TRIM21 negatively regulates the development of autoimmune diseases and inflammation in autoimmune pathogenesis. Thus, TRIM21-targeted therapy may offer a useful new strategy for treating autoimmune diseases.

## Figures and Tables

**Table 1:** SLE/SS-related SNP in *TRIM21* gene.

Location (position)*	Reference SNP ID	Nucleotide change	Genotype frequency (%)		*P* Value	Reference
5′-UTR	rs5030767		Ab(+) pSS	HC		
(4595)			(*n* = 38)	(*n* = 72)		
		C/C	60.5	83.3	0.029	[22]
		C/T	34.2	13.9		
		T/T	5.3	2.8		

Intron 1	rs7947461		Ab(+) pSS	Ab(−) pSS		
(7216)			(*n* = 39)	(*n* = 23)		
		A/A	25.6	4.3	0.028	[23]
		A/G	56.4	52.2		
		G/G	17.9	43.5		

Intron 1	rs660		SLE	HC		
(7219)			(*n* = 26)	(*n* = 29)		
		C/C	3.8	34.5	<0.0005	[20, 21]
		C/T	26.9	44.8		
		T/T	69.2	20.7		

Intron 1	rs5030768		Ab(+) pSS	HC		
(7649)			(*n* = 38)	(*n* = 72)		
		A/A	57.9	80.5	0.038	[22]
		A/G	34.2	16.7		
		G/G	7.9	2.8		

Intron 3	rs915956		Ab(+) pSS	HC		
(9571)			(*n* = 38)	(*n* = 72)		
		C/C	44.7	84.7	0.00003	[22]
		C/T	52.6	12.5		
		T/T	2.6	2.8		

3′-UTR	rs4144331		Ab(+) pSS	HC		
(12986)			(*n* = 38)	(*n* = 72)		
		C/C	71.1	87.5	0.033	[22]
		C/A	28.9	12.5		

*Position in the gene according to GenBank accession no. UO1882. UTR, untranslated region; Ab(+), anti-TRIM21 antibody-positive; pSS, primary SS, HC, healthy control; Ab(−), anti-TRIM21 antibody-negative.

**Table 2:** TRIM21-binding proteins.

Protein	Molecular function of TRIM21	Functional output	Reference
	(1) Substrate of TRIM21 E3 ligase		

IRF3	Proteasomal degradation	Suppressing IFN-*β* production	[68]
IRF7	Proteasomal degradation	Suppressing IFN-*α* production	[70]
IRF8	Transcriptional activation	Promoting IL-12p40 production	[41]
IgG	Proteasomal degradation	Neutralization of IgG-coated virion	[82]
IKK*β*	Translocation to autophagosomes	Suppressing NF-*κ*B signaling	[91]
UnpEL	Unknown	Unknown	[39]
TRIM5*α*	Translocation to cytoplasm	Unknown	[111]

	(2) Proteins supporting TRIM21 E3 activity		

Skp2	Facilitating p27 degradation	Promoting S-phase progression	[38]
Skp1	Facilitating p27 degradation	Promoting S-phase progression	[38]
Cul1	Facilitating p27 degradation	Promoting S-phase progression	[38]
*β*TrCP2	Unknown	Unknown	[38]
p62	Facilitating IRF8 degradation	Suppressing IL-12p40 production	[67]
TRIB2	Facilitating C/EBP*α* degradation	Promoting lung tumorigenesis	[85]
FADD	Facilitating IRF7 degradation	Suppressing IFN-*α* production	[71]

	(3) Proteins irrelevant to TRIM21 E3 activity		

IRF3	Stabilization	Promoting IFN-*β* production	[69]
Daxx	Unknown	Unknown	[84]
Flash	Translocating Daxx to cytoplasm	Unknown	[84]
DCP2	Facilitating decapping activity	Unknown	[86]
